# Genetic and immunological determinants of Pemphigus vulgaris: integrative analysis of HLA-DRB1 and FCGR2B variants

**DOI:** 10.1007/s00251-026-01402-5

**Published:** 2026-05-20

**Authors:** Burak Kaan Kasap, Bayram Toraman, Burçin Kurt, Hande Ermis, Deniz Aksu Arıca

**Affiliations:** 1 Department of Medical Biology, Institute of Health Sciences, Karadeniz Technical University, Trabzon, Türkiye; 2 Department of Medical Biology, Faculty of Medicine, Karadeniz Technical University, Trabzon, Türkiye; 3 Department of Biostatistics and Medical Informatics, Faculty of Medicine, Karadeniz Technical University, Trabzon, 61080 Türkiye; 4 Department of Dermatology and Venereology, Faculty of Medicine, Koç University, İstanbul, Türkiye; 5 Department of Dermatology, Faculty of Medicine, Karadeniz Technical University, Trabzon, Türkiye

**Keywords:** Pemphigus vulgaris, HLA-DRB1, FCGR2B, Immunogenetics, Bioinformatics

## Abstract

Pemphigus vulgaris (PV) is a rare autoimmune blistering disease mediated by pathogenic autoantibodies. Although both HLA and non-HLA loci contribute to disease susceptibility, their combined roles in immune regulation remain incompletely understood. This study investigated the joint contribution of HLA-DRB1 and FCGR2B variants to PV susceptibility within an integrative immunogenetic framework. Genotype data from 286 individuals (200 controls and 86 PV patients) were analyzed using bias-reduced association models, two-locus genotype combination analyses, and cumulative genetic risk modeling. Gene–gene relationships were explored through epistasis testing, while functional relevance was examined using biological annotation approaches. HLA-DRB1*04:02 and *14:01 were significantly associated with increased PV risk, whereas *11:01 and *16:01 demonstrated protective effects after multiple testing correction (q < 0.05). The FCGR2B c.671T > C (I232T) variant showed a modest effect in single-locus analyses but did not remain statistically significant after correction for multiple testing. Several two-locus genotype combinations involving FCGR2B and HLA-DRB1 were enriched among patients; however, interaction analyses supported an additive immunogenetic architecture rather than epistasis. Genetic risk modeling demonstrated improved discrimination with weighted scores, and explainable machine-learning analysis identified HLA-DRB1 as the dominant predictor, with FCGR2B contributing a secondary modulatory signal. These findings delineate an additive immunogenetic framework underlying PV susceptibility, emphasizing the central role of HLA-DRB1. Although FCGR2B did not retain independent statistical significance after multiple-testing correction, the results suggest a potential modulatory contribution within the broader immunogenetic architecture of PV.

**Supplementary Information:**

The online version contains supplementary material available at 10.1007/s00251-026-01402-5.

## Introduction

Pemphigus vulgaris (PV) is a life-threatening autoimmune blistering disease characterized by intraepithelial acantholysis, which leads to erosions of the skin and mucous membranes. The disease is mediated by pathogenic immunoglobulin G (IgG) autoantibodies that target desmoglein-3 (Dsg3) and desmoglein-1 (Dsg1), two critical cadherin-type adhesion molecules responsible for keratinocyte cohesion (Amagai et al. 2006). Disruption of desmosomal integrity results in the loss of cell–cell adhesion and subsequent blister formation. Although corticosteroids and immunosuppressive agents have significantly reduced mortality, relapses are common, and the molecular basis of disease susceptibility remains incompletely understood. Cumulative evidence supports a multifactorial etiology involving genetic, immunological, and environmental factors that contribute to immune dysregulation and autoantibody production (Didona et al. 2019).

Genetic predisposition plays a central role in PV pathogenesis. Among the identified genetic loci, human leukocyte antigen (HLA) class II alleles—especially HLA-DRB1—are the most strongly associated with disease susceptibility (Stanley and Amagai 2006). Multiple studies have confirmed that HLA-DRB1*04:02, HLA-DRB1*14:01, and HLA-DRB1*08:04 confer significant risk in different populations, including Ashkenazi Jewish, Mediterranean, and South Asian cohorts (Loiseau et al. 2000, Yan et al. 2012, Alpsoy et al. 2015). These alleles are believed to enhance antigen presentation to autoreactive CD4 + T cells, thereby promoting the loss of tolerance and the generation of pathogenic antibodies. Conversely, HLA-DRB1*07:01 and HLA-DQB1*02:02 have been described as protective alleles in some ethnic groups, suggesting population-specific immunogenetic variation (Delgado et al. 1997). Despite these consistent associations, HLA polymorphisms alone cannot fully explain disease heterogeneity or clinical variability, implying the involvement of additional non-HLA genes in modulating immune homeostasis. In this context, Fc gamma receptor IIB (*FCGR2B*) has emerged as an important candidate gene. *FCGR2B* encodes the only inhibitory Fcγ receptor on B cells and macrophages and functions as a negative regulator of B-cell activation (Nimmerjahn and Ravetch 2008). Functional polymorphisms in FCGR2B, such as the I232T variant—impair inhibitory signaling, leading to heightened B-cell responsiveness and autoantibody production (Kono et al. 2005). Altered FCGR2B expression or function has been linked to autoimmune diseases including systemic lupus erythematosus and rheumatoid arthritis (Siriboonrit et al. 2003, Blank et al. 2009), but its contribution to PV remains underexplored.

Previous studies examining the immunogenetic background of pemphigus vulgaris (PV) have generally focused on individual loci, often evaluating HLA or non-HLA genes separately. Numerous investigations have reported associations between specific HLA-DRB1 alleles and PV susceptibility across different populations (Yan et al. 2012, Baker et al. 2022). In contrast, non-HLA immune-regulatory genes, particularly FCGR2B, have been less extensively studied, despite their established roles in modulating antibody-mediated immune responses. Variants within FCGR2B have been shown to influence the inhibitory signaling of B cells and have been associated with systemic lupus erythematosus and rheumatoid arthritis (Siriboonrit et al. 2003, Blank et al. 2009). Although these findings suggest that FCGR2B may contribute to autoantibody-driven diseases, its relevance in PV remains uncertain. Moreover, studies simultaneously assessing HLA-DRB1 and FCGR2B polymorphisms in PV are scarce. Evidence from other autoimmune neurological diseases, such as Guillain–Barré syndrome, shows that combined effects of HLA class II alleles and IgG Fc receptor polymorphisms can modulate disease susceptibility (Sinha et al. 2010). However, such gene–gene relationships have not been systematically investigated in PV. A better understanding of how these genetic factors interact could clarify the complex immunological mechanisms underlying PV and support future efforts toward comprehensive, genetics-based risk assessment.

Therefore, this study aimed to systematically investigate the independent and combined effects of HLA-DRB1 and FCGR2B variants on susceptibility to pemphigus vulgaris (PV) using an integrative analytical framework that merges statistical, bioinformatic, and machine learning approaches. Genotyping data from 286 individuals were analyzed using Firth penalized logistic regression, two-locus genotype combination analyses, and gene–gene interaction modeling to characterize the underlying genetic architecture. Functional annotation and pathway mapping analyses (GO, KEGG, and STRING PPI) were additionally conducted to identify immune-related molecular networks linking these loci to PV pathophysiology.

To enhance predictive and biological interpretability, an explainable machine learning model (XGBoost with SHAP) was implemented to assess the discriminative power of the identified variants and quantify their contribution to disease risk. By integrating genetic, statistical, functional, and interpretable AI evidence, this study aims to clarify the additive and modulated immunogenetic mechanisms shaping PV susceptibility and provide a data-driven foundation for precision risk modeling in autoimmune blistering diseases.

## Materials and methods

### Study design and participants

This study was conducted as a continuation of an interdisciplinary research project supported by the Karadeniz Technical University (KTU) Scientific Research Coordination Unit (Project No: TDI-2022-10157), undertaken in collaboration among the Departments of Medical Biology, Dermatology and Venereology, and Medical Biochemistry at the KTU Faculty of Medicine. The investigation was designed as a case–control study aimed at elucidating the genetic etiology of pemphigus vulgaris (PV).

A total of 286 participants were enrolled, including 86 patients with PV and 200 healthy controls. Patients were recruited from the outpatient clinics of the Department of Dermatology and Venereology at KTU Faculty of Medicine. The diagnosis of PV was established based on characteristic clinical features, histopathological confirmation of intraepithelial acantholysis, and positive findings in direct immunofluorescence and/or enzyme-linked immunosorbent assay (ELISA) for desmoglein (Dsg) antibodies. Patients aged between 21 and 81 years with no concomitant autoimmune diseases were eligible for inclusion.

The control group consisted of healthy volunteers with no history of autoimmune or dermatological disorders, recruited from the same clinical setting and geographic catchment area. Demographic variables (age and sex) were recorded at recruitment; however, these variables were not included in the genetic regression models analyzed in the present study. All participants provided written informed consent prior to enrollment. From each subject, 5–9 mL of peripheral blood was collected into ethylenediaminetetraacetic acid (EDTA) tubes for genomic DNA extraction and subsequent genotyping.

The study protocol was approved by the Karadeniz Technical University Faculty of Medicine Ethics Committee (Approval No: 2022/51) and conducted in accordance with the ethical principles of the Declaration of Helsinki.

Baseline demographic characteristics of cases and controls are presented in Table 1. No statistically significant differences were observed between groups in age (*p* = 0.854) or sex distribution (*p* = 0.501).

**Table 1:** Baseline demographic characteristics of patients with pemphigus vulgaris (PV) and controls

Variable	PV (*n* = 86)	Controls (*n* = 200)	*p*-value
**Age**,** years (mean ± SD)**	52.3 ± 14.3	52.5 ± 15.9	0.854¹
**Sex**,** n (%)**			0.501²
**Female**	42 (48.8%)	108 (54.0%)	
**Male**	44 (51.2%)	92 (46.0%)	

^1^ Wilcoxon rank-sum test

^2^ Pearson’s Chi-square test with Yates’ continuity correction

### Genotyping and variant classification

Genomic DNA was extracted from peripheral blood leukocytes using the high-salt (salting-out) precipitation method. DNA purity and concentration were assessed spectrophotometrically (NanoDrop^®^ 2000 UV–Vis, Thermo Fisher Scientific), and working solutions were prepared at 50 ng/µL.

Genotype quality control procedures included verification of amplicon specificity by agarose gel electrophoresis, inspection of Sanger sequencing chromatograms for peak clarity, and alignment with the FCGR2B reference sequence to ensure unambiguous base calling. Samples with insufficient sequencing quality would have been re-amplified and re-sequenced. In the present dataset, all samples yielded definitive genotype calls. Genotype completeness for FCGR2B was 100% in both cases and controls, with no missing data observed. No samples met predefined exclusion criteria.

#### FCGR2B genotyping

The exon 5 region of the FCGR2B gene, which contains the functional c.671 T > C (I232T) variant, was amplified by a two-step polymerase chain reaction (PCR) approach due to high sequence homology with its paralog FCGR2C. Primers were designed using Primer3 and validated by in silico PCR in the UCSC Genome Browser to ensure locus specificity. Long PCR amplification was performed using the GoTaq^®^ G2 Flexi DNA polymerase (Promega, USA), followed by nested PCR under optimized thermal cycling conditions. PCR products were verified by 2% agarose gel electrophoresis and purified enzymatically using the ExS-Pure™ PCR Purification Kit (NimaGen, Netherlands).

The primer sequences and expected amplicon sizes used for FCGR2B amplification and sequencing are provided in Appendix Table 4.

This amplification strategy was designed to minimize potential interference from highly homologous FCGR paralogs, particularly FCGR2C, thereby ensuring locus-specific amplification and accurate genotyping of the FCGR2B I232T variant.

Purified amplicons were subjected to Sanger sequencing using the BigDye™ Terminator v3.1 Cycle Sequencing Kit (Thermo Fisher Scientific) and analyzed on an ABI 3130 Genetic Analyzer. Sequence chromatograms were examined using Chromas 2.6.6 and Sequencing Analyser 5.3.1 software. Genotypes were assigned by alignment with the FCGR2B reference sequence (NCBI RefSeq: NG_011467.1).

#### HLA-DRB1 typing

HLA-DRB1 genotyping was performed using the PCR–sequence-specific oligonucleotide probe (PCR-SSOP) Luminex method with the One Lambda LABType^®^ SSO DRB1 Typing Kit (Thermo Fisher Scientific, USA).

This assay involves amplification of target HLA regions followed by hybridization with allele-specific oligonucleotide probes covalently bound to fluorescent microbeads. Hybridization signals were detected by Luminex LABScan 3D (One Lambda, USA), and allele assignment was performed using HLA Fusion software according to the manufacturer’s recommendations.

All reagents, primers, and equipment were sourced from validated commercial suppliers, and all genotyping procedures were conducted at the Karadeniz Technical University Medical Biology and Hematology Laboratories following standard molecular protocols.

### Statistical, bioinformatic, and machine learning analyses

All statistical, bioinformatic, and machine learning analyses were performed using R software (version 4.3.2; R Foundation for Statistical Computing, Vienna, Austria). Classical statistical models (Firth logistic regression, allele/genotype association, and genetic risk score analysis) were combined with bioinformatic functional annotation and pathway mapping analyses (STRING, KEGG, and GO analyses) and predictive modeling approaches (XGBoost with SHAP interpretability). A two-sided *p* < 0.05 was considered statistically significant for all inferential tests, and model performance was evaluated using ROC–AUC, sensitivity, specificity, precision, and F1 metrics.

A. ***Genotype–Phenotype Association Analysis***
  Descriptive statistics were calculated to summarize demographic and genotypic distributions. Differences between pemphigus vulgaris (PV) patients and controls were evaluated using Fisher’s exact or chi-square tests (Agresti 2013).
  This component of the analysis followed a candidate gene association study (CGAS) framework, assessing the relationship between HLA-DRB1 and FCGR2B loci and disease susceptibility.
  Single-locus association analyses for FCGR2B c.671 T > C (I232T) and HLA-DRB1 alleles were performed under dominant, recessive, and additive genetic models using logistic regression.
  Given the moderate sample size and presence of rare genotypes, Firth’s penalized likelihood logistic regression was applied to reduce small-sample bias and improve parameter estimation (Firth 1993, Heinze and Schemper 2002). Effect sizes were reported as odds ratios (ORs) with 95% confidence intervals (CIs).
  Multiple testing correction was performed using the Benjamini–Hochberg false discovery rate (FDR) procedure (Benjamini and Hochberg 1995). The correction was applied across the HLA-DRB1 allele-level association tests that passed the predefined frequency threshold (≥ 3% allele frequency), corresponding to 11 allele-specific hypothesis tests.
  Allele counts and group-specific frequencies for all HLA-DRB1 variants are provided in Appendix Table 5. Alleles observed fewer than three times in either group were not interpreted individually in regression analyses in order to avoid unstable or inflated effect size estimates.
B. ***Two-Locus Genotype Combination Analysis and Epistasis Modeling***
  Population-based analyses were conducted to evaluate combined genetic effects on pemphigus vulgaris (PV) susceptibility. For HLA-DRB1, locus-level analyses were performed by comparing allele frequencies and allele-pair (genotype) distributions derived directly from the two reported alleles (A1 and A2) per individual. Allele-pair categories were generated by concatenating A1 and A2 (e.g., “04:02–14:01”) and tabulated by phenotype group. Rare categories (<1% frequency) were excluded to reduce sparse-cell instability and to improve the robustness of population-based genotype combination analyses and haplotype inference approaches (Excoffier and Slatkin 1995; Lake et al. 2003; Schaid et al. 2002). 
  To assess combined effects across loci located on different chromosomes, we constructed two-locus genotype (allele) combinations between FCGR2B (coded as TT/CT/CC and also as C-allele carrier vs. TT where specified) and HLA-DRB1 categories. Distributions between cases and controls were evaluated using Fisher’s exact test, and significant combinations were visualized using frequency bar plots (*ggplot2*) (Wickham 2016). For computational efficiency and compact visualization in score-based two-locus analyses, alleles were numerically encoded (FCGR2B: 1 = C, 2 = T; HLA-DRB1 alleles coded according to factor-level ordering in R). This numeric encoding was used solely for statistical computation and figure labeling, and not for biological interpretation. The complete code-to-allele mapping is provided in Appendix Table 6.
  In addition to two-locus genotype combination analyses, epistasis modeling was performed to formally test for gene–gene interactions between HLA-DRB1 and FCGR2B loci. An interaction term was incorporated into multivariable logistic regression models to evaluate potential non-additive effects (Cordell 2002, Cordell 2009). This integrative approach allowed assessment of whether the combined effects of these loci deviated from an additive model in contributing to PV susceptibility.
C. ***Genetic Risk Modeling and Explainable AI Framework***
  To evaluate the cumulative effect of genetic variants, both unweighted and weighted Genetic Risk Scores (GRS) were computed using R (tidyverse, DescTools, Hmisc, and pROC packages). The GRS incorporated the significant variants identified in the association analyses. Specifically, the risk alleles HLA-DRB1*04:02 and HLA-DRB1*14:01, together with the FCGR2B C allele, were included as susceptibility variants. Protective alleles (HLA-DRB1*11:01 and HLA-DRB1*16:01) were evaluated separately within the regression modeling framework.
  For the weighted GRS, individual genotypes were coded as 0, 1, or 2 according to the number of risk alleles carried, and each genotype count was multiplied by the corresponding β-coefficients derived from Firth penalized logistic regression models (Heinze and Schemper 2002). The weighted GRS was obtained by summing these weighted contributions across loci. The unweighted GRS was calculated as the total number of risk alleles carried by each individual across loci, following established polygenic modeling frameworks (Chatterjee et al. 2016).
  Model performance was evaluated using complementary discrimination and calibration metrics. Discrimination was assessed using the area under the receiver operating characteristic curve (ROC–AUC) implemented in the pROC package (Robin et al. 2011), with 95% confidence intervals estimated using the DeLong method. Model calibration was quantified using Somers’ D and Brier scores calculated with the Hmisc and DescTools packages (Steyerberg et al. 2010).
  To capture potential non-linear and higher-order gene–gene relationships, an explainable artificial intelligence (XAI) framework was additionally applied. An Extreme Gradient Boosting (XGBoost) classifier (Chen and Guestrin 2016) was trained using genetic predictors (HLA_risk_allele, FCGR2B_C_carrier, interaction_term, and GRS_unweighted). The dataset was randomly partitioned into training and testing subsets using a 70:30 split, and model performance was evaluated exclusively on the held-out test dataset. Class imbalance between cases and controls was addressed using the scale_pos_weight parameter. The main XGBoost hyperparameter settings used in the study are provided in Appendix Table 7.
  To further assess model stability and potential overfitting, a learning curve analysis was performed by training the model on progressively larger subsets of the training dataset and evaluating performance on an internal validation subset. Training and validation AUC values were recorded across increasing training sample sizes to examine the relationship between dataset size and predictive performance.
  To interpret model predictions, SHapley Additive exPlanations (SHAP) values were computed to quantify the contribution of each predictor to disease classification (Lundberg and Lee 2017). This explainability framework enabled direct comparison between machine-learning–derived feature importance and classical statistical findings derived from regression and GRS-based analyses.
D. ***Functional Annotation and Pathway Mapping***
  To elucidate the potential biological mechanisms underlying the observed genetic associations, functional annotation and protein–protein association analyses were performed. The identified genes (*HLA-DRB1* and *FCGR2B*) were mapped to Gene Ontology (GO) categories and Kyoto Encyclopedia of Genes and Genomes (KEGG) pathways using the *clusterProfiler* R package (Yu et al. 2012) with the *org.Hs.eg.db* human genome annotation database (Carlson 2019). This approach provides GO Biological Process (BP), Molecular Function (MF), and Cellular Component (CC) descriptors (Ashburner et al. 2000), as well as KEGG pathway mappings (Kanehisa and Goto 2000), characterizing the well-established immunological roles of these genes.
  Because the analysis was conducted on two genes, no statistical overrepresentation (hypergeometric) test was performed. Instead, GO and KEGG terms were retrieved through functional annotation using curated ontology mappings implemented in *clusterProfiler*. The reported GO and KEGG categories therefore represent biological descriptors of known gene functions rather than statistically enriched pathways.
  Functional associations between HLA-DRB1 and FCGR2B were examined using a protein–protein association network derived from the STRING database (version 12.0) (Szklarczyk et al. 2023). The analysis was implemented via the STRINGdb R interface (Franceschini et al. 2013), which integrates multiple evidence channels—including co-expression, curated pathway databases, text mining, and experimental annotations—to infer functional relationships rather than direct physical interactions. Protein association data were filtered at a medium-to-high confidence score (≥ 0.4), consistent with recommended practice in network-based functional annotation. Network significance was assessed using STRING’s observed-to-expected edge statistics, which evaluate functional connectivity at the network level but do not imply biochemical interaction. Network visualization and mapping were performed in R using *igraph* (Csardi and Nepusz 2006) and *ggraph* (Pedersen 2022) to illustrate functional linkage between the two immune-related proteins.

## Results and findings

### Genotype–phenotype association results

Genotype and allele frequency distributions for HLA-DRB1 and FCGR2B were analyzed to identify potential associations with pemphigus vulgaris (PV) susceptibility. The genotype distribution for FCGR2B c.671T > C (I232T) was as follows: in the control group (*n* = 200), TT = 167 (83.5%), CT = 29 (14.5%), and CC = 4 (2.0%); in the disease group (*n* = 86), TT = 65 (75.6%), CT = 18 (20.9%), and CC = 3 (3.5%). Hardy–Weinberg equilibrium (HWE) testing in the control group yielded χ² *p* = 0.0539 and exact *p* = 0.0695, indicating that FCGR2B genotype distributions were consistent with Hardy–Weinberg equilibrium.

As part of the candidate gene association study (CGAS) framework, single-locus analyses were conducted under dominant, recessive, and additive genetic models for both loci to evaluate potential inheritance patterns and their contributions to PV susceptibility.

Allele-based association analysis revealed multiple HLA-DRB1 alleles significantly associated with PV after Benjamini–Hochberg FDR correction. The *04:02* allele (OR = 30.10, 95% CI = 15.50–61.40, q < 0.001) and *14:01* allele (OR = 5.66, 95% CI = 2.96–11.10, q < 0.001) were identified as the major risk variants, while *16:01* (OR = 0.05, 95% CI = 0.0004–0.34, q = 0.001) and *11:01* (OR = 0.36, 95% CI = 0.14–0.82, q = 0.037) showed protective effects (Table 2).

**Table 2:** Allele-based associations of HLA-DRB1 with pemphigus vulgaris

*Allele*	*OR*	*95% CI*	*p-Value*	*FDR (q)*	*Interpretation*
04:02	30.10	15.50–61.40	< 0.001	< 0.001	Strong risk
14:01	5.66	2.96–11.10	< 0.001	< 0.001	Risk
16:01	0.05	0.0004–0.34	< 0.001	0.001	Protective
11:01	0.36	0.14–0.82	0.013	0.037	Protective

These findings were consistent across both Fisher’s exact tests and Firth penalized logistic regression models, supporting the robustness of the observed HLA-DRB1 associations.

**Fig. 1:**
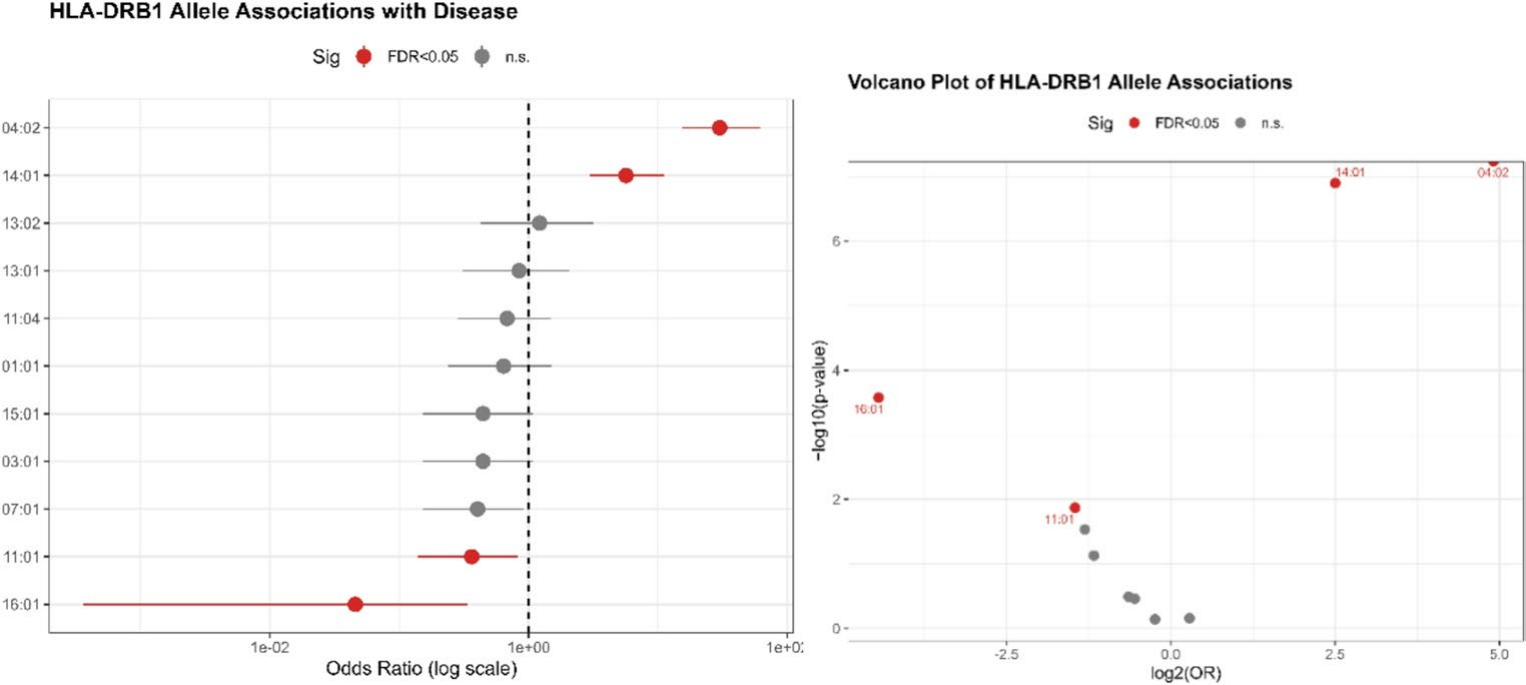
Allele-based associations of HLA-DRB1 with PV. *Note*: The left panel (forest plot) presents odds ratios (ORs) and 95% confidence intervals for individual HLA-DRB1 alleles, derived from Firth penalized logistic regression. The right panel (volcano plot) displays − log₁₀(p) versus log₂(OR), highlighting the magnitude and statistical significance of each allele

Visual representations of the HLA-DRB1 association results are provided in Fig. 1 (*left-*forest plot) and Fig. 1 (*right*-volcano plot).

The forest plot clearly demonstrates the strong positive effect of the HLA-DRB1*04:02 and HLA-DRB1*14:01 alleles, while the volcano plot highlights their high significance levels and magnitude of risk contribution relative to other alleles. Together, these results underscore HLA-DRB1 as the dominant immunogenetic determinant of PV susceptibility.

In contrast, the FCGR2B c.671 T > C (I232T) variant did not reach statistical significance in either Fisher’s exact or Firth logistic regression analysis under the dominant genetic model (OR = 1.63, 95% CI = 0.83–3.15, q = 0.15). Additive (OR = 1.51, q = 0.15) and recessive (OR = 1.83, q = 0.41) models yielded similar non-significant trends, suggesting a consistent but modest risk direction for the C allele across inheritance patterns. Although the direction of effect suggested a possible risk increase among C-allele carriers, the result did not survive correction for multiple testing. Given its non-significance, FCGR2B results are presented in Appendix A (Table 8 and Fig. 7).

### Two-locus genotype combination analysis and epistasis modeling results

Comprehensive two-locus genotype combination analyses revealed distinct patterns of association between HLA-DRB1 and FCGR2B variants in relation to pemphigus vulgaris (PV) susceptibility. Combination-level findings identified both higher-risk and potentially protective genotype profiles across the two loci. Formal epistasis modeling further clarified the relative contributions of each gene, indicating that their effects were primarily additive rather than non-additive.

**Fig. 2:**
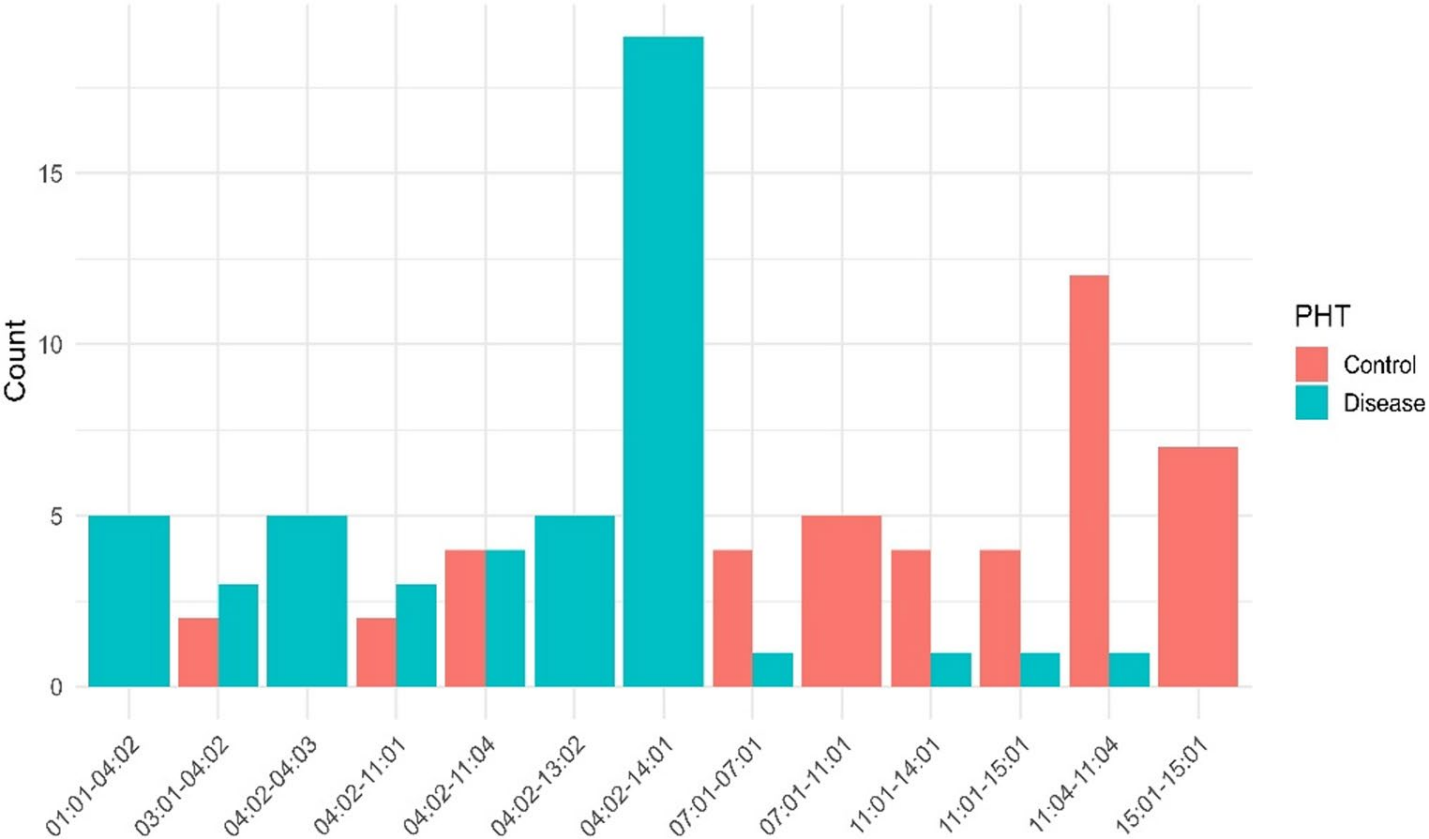
Distribution of common HLA-DRB1 genotype combinations (diplotypes) in pemphigus vulgaris (PV) patients and controls. *Note: *Bar plot showing the relative frequency of major HLA-DRB1 genotype combinations in PV patients and controls. Genotype combinations containing HLA-DRB1*04:02 and HLA-DRB1*14:01 are more frequent among PV patients (teal), whereas combinations including HLA-DRB1*11:01 or HLA-DRB1*16:01 occur more frequently in controls (red), consistent with their potential protective effects

Among HLA-DRB1 genotype combinations (diplotypes), several allele combinations showed strong disease associations. The most frequent risk combinations were DRB1*04:02–DRB1*14:01 (frequency = 0.168, *p* < 0.001, q < 0.01) and DRB1*14:01–DRB1*04:02 diplotypes (frequency = 0.155, *p* < 0.001, q < 0.01), both conferring markedly elevated risk consistent with single-allele results. Conversely, genotype combinations containing DRB1*16:01 or DRB1*11:01 exhibited protective effects (*p* = 0.012 and *p* = 0.035, respectively). The distribution of common HLA-DRB1 genotype combinations (diplotypes) in cases and controls is illustrated in Fig. 2, emphasizing the overrepresentation of 04:02–14:01 among PV cases.

**Fig. 3:**
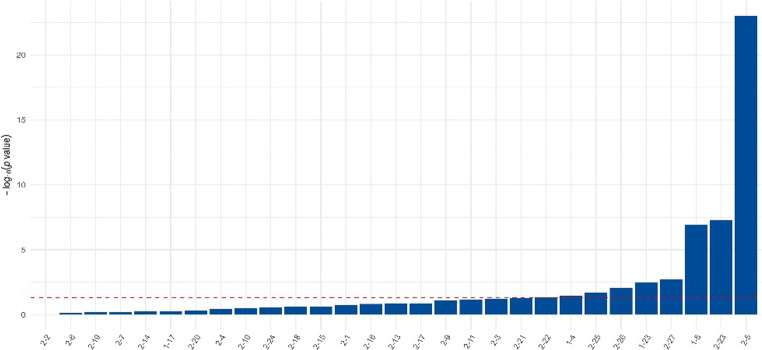
Association of combined FCGR2B–HLA-DRB1 genotype combinations with pemphigus vulgaris. *Note:* Each bar represents a distinct two-locus genotype combination ordered by −log10(p). The dashed red line indicates the nominal significance threshold (*p* = 0.05). Taller bars correspond to genotype combinations showing stronger statistical association with disease risk, whereas shorter bars indicate weaker or non-significant associations

Two-locus genotype combination analysis integrating HLA-DRB1 and FCGR2B identified several combinations significantly associated with PV. The strongest risk multi-locus combination was FCGR2B–HLA-DRB1 (C–HLA-DRB1*04:02) (frequency = 11.6%, score = 10.04, *p* = 9.96 × 10⁻²⁴), followed by T–HLA-DRB1*12:01 (*p* = 4.95 × 10⁻⁸) and additional combinations involving HLA-DRB1*04:02 (*p* = 1.22 × 10⁻⁷). For clarity, numeric codes used in the analysis were mapped to standard allele nomenclature (see Appendix Table 6). Conversely, specific two-locus genotype combinations involving the FCGR2B T allele and HLA-DRB1*13 variants (HLA-DRB1*13:02, HLA-DRB1*13:03, and HLA-DRB1*13:05) were associated with negative score statistics (*p* < 0.05), suggesting a protective direction of effect (Fig. 3).

These findings indicate a coordinated contribution of antigen presentation (HLA-DRB1) and Fc receptor regulation (FCGR2B) pathways, consistent with a polygenic model of autoimmune susceptibility in PV.

**Fig. 4:**
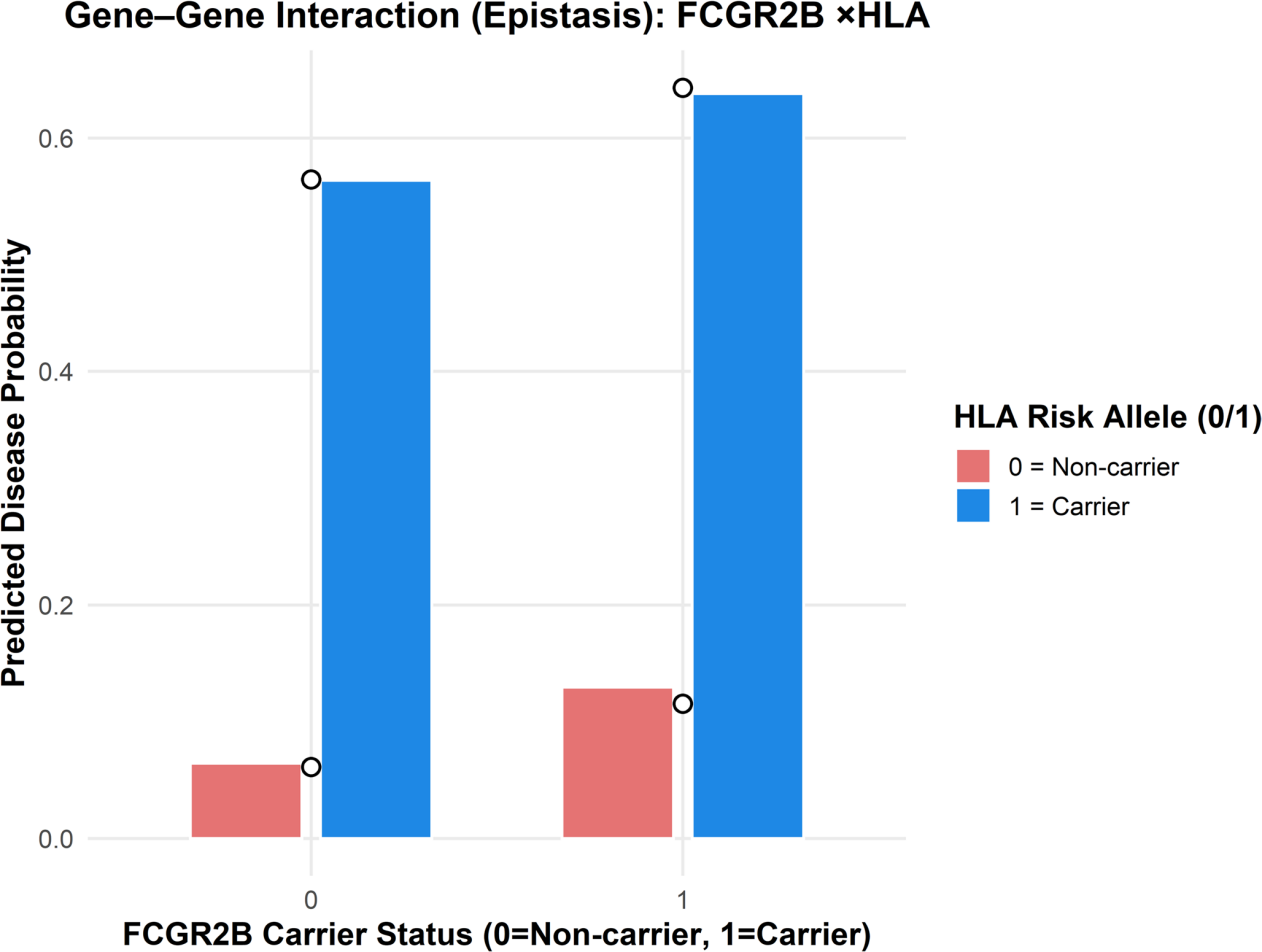
Predicted and observed disease probabilities for the interaction between FCGR2B and HLA-DRB1 risk alleles. *Note:* Carriers of HLA_risk_alleles (blue bars) show markedly elevated disease probabilities regardless of FCGR2B status, indicating a dominant HLA effect. The FCGR2B_C_carrier variable exerts a small, non-significant additive effect. Open circles represent observed disease rates, closely aligned with model predictions, indicating good calibration and absence of epistatic interaction

Epistasis modeling (Fig. 4) demonstrated a dominant main effect of HLA-DRB1 risk alleles, with carriers showing a ninefold increase in predicted disease probability (6% → 56%). Although FCGR2B_C_carrier status was associated with a minor additive increase (11–13%), the interaction_term (FCGR2B × HLA-DRB1) was not statistically significant (*p* = 0.57), indicating additive rather than multiplicative effects. Predicted probabilities closely matched observed disease rates, reflecting good model calibration.

To further validate the observed genetic patterns, multivariable Firth logistic regression models were conducted integrating FCGR2B and HLA-DRB1 predictors (Appendix Table 9). The HLA-only model (Model 2) exhibited strong discriminatory ability (AUC = 0.80), confirming the dominant contribution of HLA-DRB1 to PV susceptibility. The combined model including both genes (Model 3) achieved a comparable performance (AUC = 0.81), while inclusion of the interaction_term (FCGR2B × HLA) did not significantly improve fit (*p* = 0.570, FDR (q) = 0.410).

These regression-based findings further support an additive rather than epistatic relationship between the loci, consistent with the two-locus genotype combination and interaction analyses described above.

### Genetic risk modeling and XAI results

To quantify the cumulative impact of HLA-DRB1 and FCGR2B variants on disease susceptibility, both unweighted and weighted Genetic Risk Scores (GRS) were constructed. The unweighted GRS, calculated as the total number of susceptibility alleles carried by each individual, showed a strong association with disease risk (OR = 9.03, 95% CI = 5.33–16.45, *p* < 0.001). Each additional risk allele substantially increased disease odds, demonstrating a clear cumulative genetic effect. The model exhibited good discriminative ability with an AUC of 0.82 (95% CI: 0.77–0.87), accompanied by a Somers’ D value of 0.64 and a Brier score of 0.15, indicating satisfactory discrimination and calibration (Table 3).

**Table 3:** Predictive performance of unweighted and weighted genetic risk score models

*Model*	*OR (95% CI)*	*p-value*	*AUC (95% CI)*	*Somers’ D*	*Brier*
***Unweighted GRS***	9.03 (5.33–16.45)	< 0.001	0.82 (0.77–0.87)	0.64	0.15
***Weighted GRS***	2.74 (2.24–3.44)	< 0.001	0.89 (0.85–0.94)	0.78	0.10

The weighted GRS, which incorporated regression-based β-coefficients to account for variant-specific effect sizes, demonstrated improved predictive performance compared with the unweighted score (OR = 2.74, 95% CI = 2.24–3.44, *p* < 0.001). The weighted model showed strong discrimination with an AUC of 0.89 (95% CI: 0.85–0.94), accompanied by a Somers’ D value of 0.78 and a Brier score of 0.10, indicating strong discriminative ability and good calibration (Table 3). These findings suggest that incorporating variant-specific effect sizes enhances the predictive capacity of genetic risk modeling.

To further explore potential non-linear genetic relationships, an Explainable Artificial Intelligence (XAI) framework was applied using an XGBoost classifier. The model incorporated four predictors: HLA_risk_allele, FCGR2B_C_carrier, interaction_term, and GRS_unweighted. The optimized model achieved strong predictive performance, with an AUC of 0.88 (95% CI: 0.82–0.95), accuracy of 0.84, sensitivity of 0.95, specificity of 0.79, and an F1-score of 0.75, indicating robust classification performance.

To further assess model stability and potential overfitting, a learning curve analysis was performed by training the model on progressively larger subsets of the training dataset and evaluating performance on an internal validation subset, as shown in Appendix A (Fig. 8). The learning curve analysis indicated that the XGBoost model achieved comparable training and validation AUC values across increasing training sample sizes, suggesting a balanced bias–variance trade-off without clear evidence of overfitting. Although variability was higher at smaller training sizes, performance stabilized as the dataset increased, indicating improved model generalization and robustness. 

**Fig. 5:**
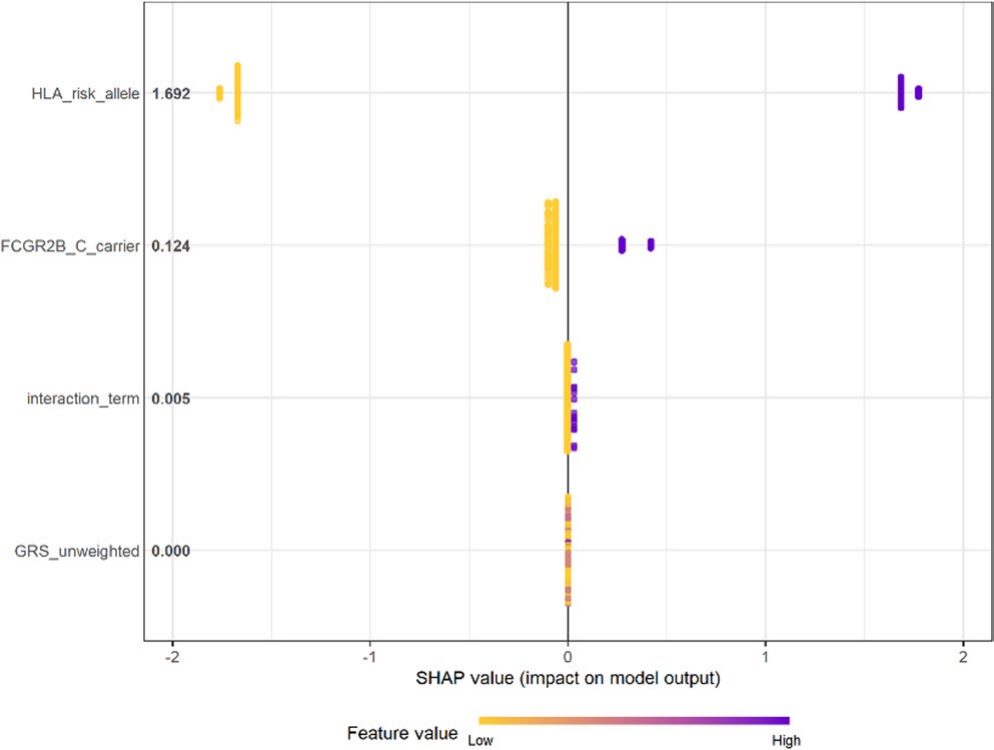
SHAP summary plot showing the relative contribution of genetic predictors to pemphigus vulgaris classification. *Note:* *HLA_risk_allele* exhibited the highest mean |SHAP| value (1.69), indicating the dominant influence on model output. *FCGR2B_C_carrier* showed a smaller but detectable contribution (0.12), whereas the *interaction_term* had a minimal effect (0.01). The contribution of *GRS_unweighted* was negligible. Warmer colors indicate higher feature values (greater disease risk), while cooler tones represent lower values

Model interpretability was assessed using SHapley Additive exPlanations (SHAP) (Fig. 5). The SHAP summary plot identified HLA_risk_allele as the dominant predictor (mean |SHAP| = 1.69), followed by FCGR2B_C_carrier (0.12). The interaction_term showed minimal contribution (0.01), and the contribution of GRS_unweighted was negligible. These results highlight the predominant role of HLA-DRB1 risk alleles in disease classification, with FCGR2B acting as a secondary modulator.

Overall, the explainability analysis confirmed that the strong predictive signal of the machine learning model was primarily driven by the HLA-DRB1 risk allele, consistent with the findings from the regression-based association analyses and two-locus genotype combination results.

Figure 5 further highlights the predominant role of HLA-DRB1 in disease classification, with FCGR2B acting as a secondary genetic modulator. The minimal contribution of the interaction term suggests limited evidence for strong non-linear gene–gene interactions in the XGBoost model. Overall, these findings are consistent with the regression-based association analyses and two-locus genotype combination results, reinforcing the central contribution of HLA-DRB1 risk alleles to disease susceptibility.

### Functional annotation and pathway mapping results

**Fig. 6:**
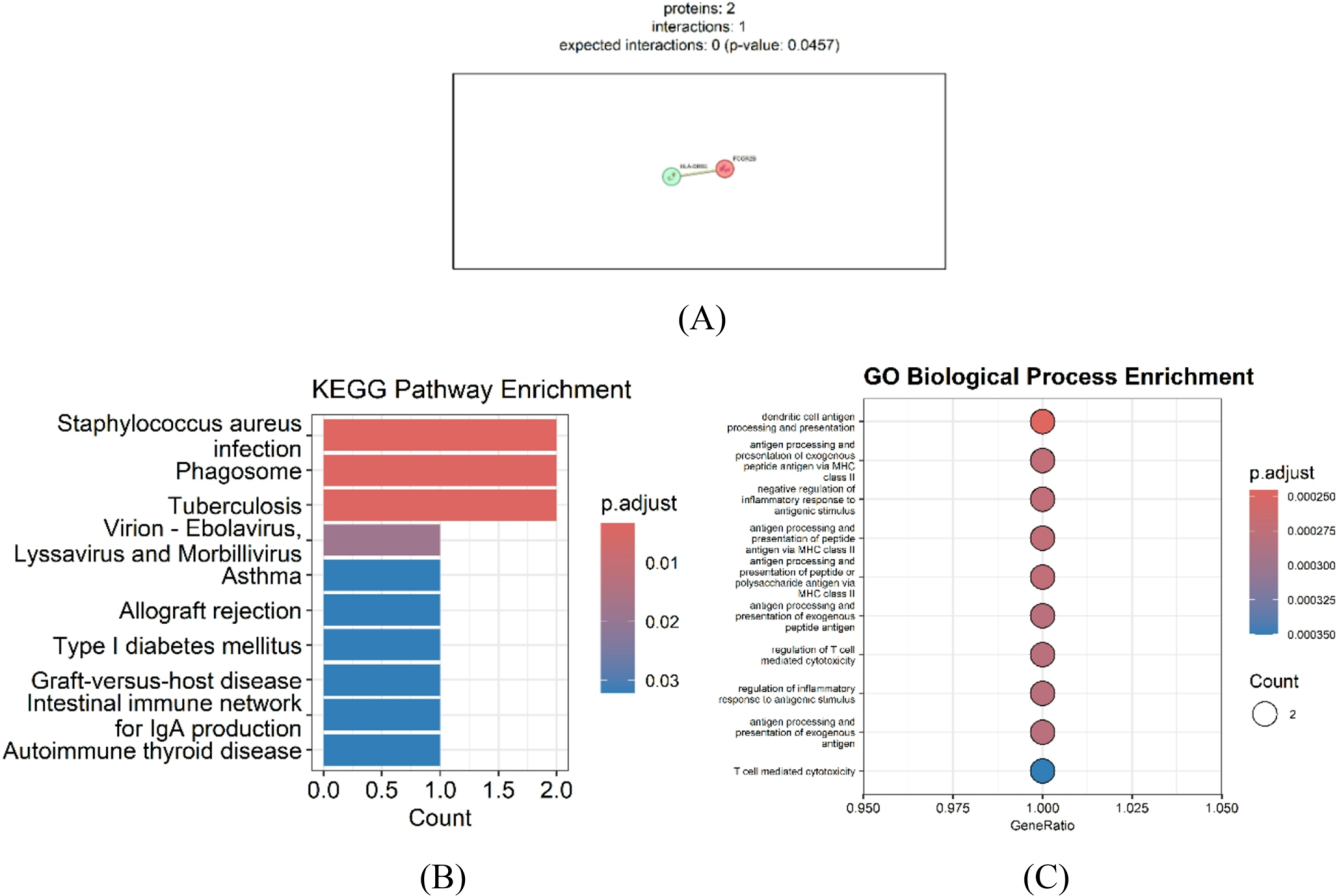
Functional annotation and pathway mapping analyses for HLA-DRB1 and FCGR2B. (**A**) STRING-based protein–protein interaction (PPI) network illustrating a functional (non-physical) association between *HLA-DRB1* and *FCGR2B*. The link reflects integrated evidence from shared immune pathways rather than direct physical binding (expected interactions = 0, *p* = 0.046). (**B**) KEGG pathway mapping highlighting immune-related pathways associated with *HLA-DRB1* and *FCGR2B* (e.g., Staphylococcus aureus infection, phagosome, tuberculosis, asthma, autoimmune thyroid disease). These pathways reflect curated functional annotations rather than statistical enrichment. (**C**) GO Biological Process (BP) annotation emphasizing antigen processing and presentation, Fc-receptor signaling, and regulation of adaptive immune responses. These terms represent ontology-based functional descriptors of *HLA-DRB1* and *FCGR2B*, not enrichment statistics

**Fig. 7:**
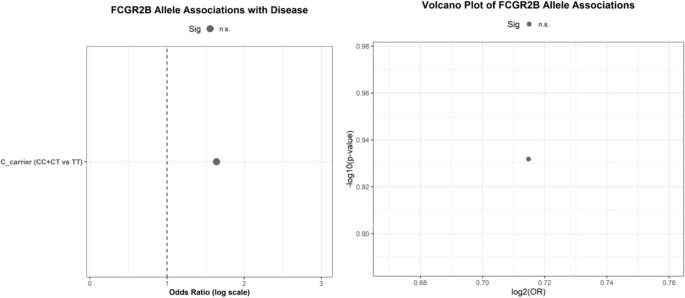
Forest and volcano plots illustrating FCGR2B allele associations with PV. *Note*. The forest plot (left) displays odds ratios (log scale) for the dominant genetic model (C-carrier: CC + CT vs. TT), while the volcano plot (right) shows the corresponding –log₁₀(p) versus log₂(OR) distribution

**Fig. 8:**
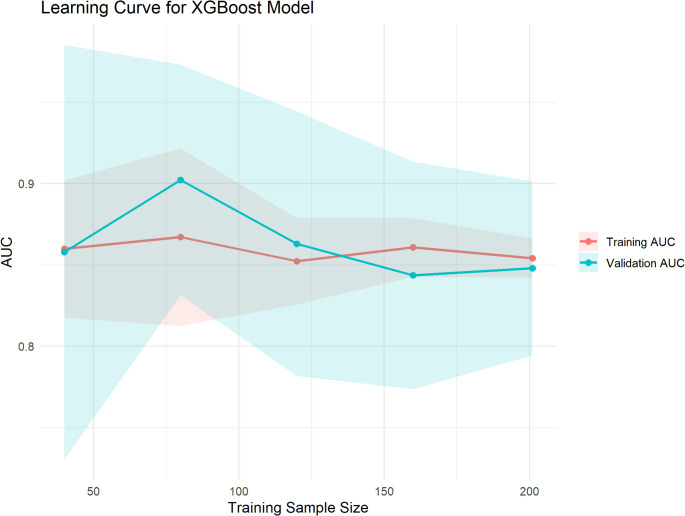
Learning curve for the XGBoost model. *Note*. The red line represents training AUC and the turquoise line represents validation AUC across progressively larger subsets of the training data. Shaded areas indicate variability across resampling iterations. The relatively small gap between the training and validation curves suggests stable model behavior and no clear evidence of severe overfitting. Although variability is greater at smaller training sample sizes, performance stabilizes as the dataset increases, indicating improved model generalization and robustness

Functional annotation and protein–protein association analyses were performed to elucidate the biological significance of the identified genetic associations. The STRING protein association network (Fig. 6A) indicated a statistically supported functional link between *HLA-DRB1* and *FCGR2B* (expected interactions = 0; *p* = 0.046). This connection reflects shared immune pathways rather than direct physical binding and suggests coordinated involvement of antigen presentation (*HLA-DRB1*) and Fc-receptor–mediated regulation (*FCGR2B*). These findings support a complementary functional relationship between the two immune genes, consistent with the additive effects observed in the genetic and epistasis analyses.

KEGG pathway mapping (Fig. 6B) highlighted well-established immune pathways associated with *HLA-DRB1* and *FCGR2B*. The mapped pathways included Staphylococcus aureus infection, phagosome, tuberculosis, asthma, type I diabetes mellitus, graft-versus-host disease, and autoimmune thyroid disease. These pathways converge on mechanisms central to antigen uptake, phagocytosis, and MHC class II–mediated signaling, reflecting the known immunological roles of the two genes. As the analysis was based on two genes, these results represent curated pathway annotation rather than statistical enrichment.

GO Biological Process (BP) annotation (Fig. 6C) further underscored biological processes relevant to antigen presentation, Fc-receptor signaling, and adaptive immune activation. Key annotated terms included antigen processing and presentation, regulation of immune effector processes, and positive regulation of adaptive immune responses. These annotations highlight the complementary immune functions of *HLA-DRB1* and *FCGR2B*, consistent with their coordinated roles suggested by the genetic and interaction analyses.

Collectively, these analyses suggest that HLA-DRB1 and FCGR2B contribute to complementary immunogenetic mechanisms integrating antigen recognition and inhibitory FcγRIIb signaling. This interpretation is consistent with the regression, two-locus genotype combination, and XAI-based findings, which support primarily additive genetic effects in PV susceptibility.

## Discussion

This integrative study combines statistical genetics, two-locus genotype combination modeling, functional annotation, and explainable machine learning to investigate how HLA-DRB1 and FCGR2B variants contribute, primarily in an additive manner, to susceptibility to pemphigus vulgaris (PV). Consistent with previous immunogenetic studies, HLA-DRB1 emerged as the dominant genetic determinant, with 04:02 and 14:01 conferring markedly increased risk and 11:01 and 16:01 demonstrating protective effects. These findings align with well-established associations in Mediterranean, Middle Eastern, and South Asian populations, where HLA-DRB1*04 and HLA-DRB1*14 allele groups have been repeatedly identified as major PV-predisposing variants (Loiseau et al. 2000, Yan et al. 2012, Alpsoy et al. 2015).

In our previous study using the same cohort, we investigated HLA-DRB1–related genetic susceptibility to pemphigus vulgaris and explored a hypothesis-generating framework linking genetic predisposition with potential environmental triggers through structural and computational analyses of vector-derived cadherin mimicry (Toraman et al. 2026). Building on these findings, the present study extends the analytical framework by incorporating cumulative genetic risk modeling and explainable machine learning approaches. In contrast to the previous work, which focused primarily on immunogenetic associations and structural plausibility of environmental mimicry mechanisms, the current study evaluates the combined contribution of HLA-DRB1 and FCGR2B variants using multi-locus genetic analysis and predictive modeling. This complementary strategy allows a more comprehensive assessment of additive genetic risk and potential non-linear relationships among susceptibility variants.

The observed protective effect of DRB1*11 and DRB1*16 alleles is consistent with immunogenetic models suggesting that certain HLA class II variants exhibit suboptimal peptide-binding conformations, resulting in reduced activation of autoreactive CD4⁺ T cells (Delgado et al. 1997).

Importantly, our results extend current knowledge by demonstrating that FCGR2B, although not independently significant after correction, contributes to PV susceptibility as a modulatory locus. The distribution and effect size of the c.671 T > C (I232T) variant paralleled findings in systemic lupus erythematosus and rheumatoid arthritis, where reduced inhibitory FcγRIIb signaling enhances autoreactive B-cell survival (Siriboonrit et al. 2003, Blank et al. 2009). While earlier PV studies rarely incorporated FCGR2B, our results show that this locus subtly modulates risk in combination with HLA, a pattern consistent with autoimmune diseases involving antibody-mediated pathology.

The lack of a strong independent effect for FCGR2B supports the interpretation that this locus may act as a modulatory rather than a primary determinant of autoimmune susceptibility, a role that is consistent with observations in systemic lupus erythematosus, rheumatoid arthritis, and other autoantibody-mediated disorders. Impaired FcγRIIb signaling alone is unlikely to initiate pathology but may amplify autoreactive B-cell responses in the presence of stronger upstream drivers such as HLA class II risk alleles. In this context, our findings suggest a framework in which HLA-DRB1 variants represent the primary genetic determinants of PV susceptibility, whereas FCGR2B may contribute a secondary modulatory effect. This interpretation highlights the importance of evaluating both loci within an integrated analytical framework while avoiding over-interpretation of individual gene effects. Importantly, Hardy–Weinberg equilibrium (HWE) testing in the control group did not demonstrate a statistically significant deviation for FCGR2B (χ² *p* = 0.0539; exact *p* = 0.0695). Given the absence of HWE distortion, the 100% genotype call rate, and rigorous sequence-based validation procedures, systematic technical artifacts are unlikely to have influenced the observed association patterns.

The locus-specific amplification strategy, chromatogram inspection, and alignment with the FCGR2B reference sequence further minimize the possibility of paralog interference or misclassification, which can be a concern within the FCGR genomic region. Therefore, the modest effect estimates observed for FCGR2B likely reflect biological variability rather than analytical bias.

Nevertheless, given the modest effect size and the absence of independent statistical significance after correction, FCGR2B findings should be interpreted as suggestive modulatory signals rather than definitive susceptibility determinants. Replication in independent cohorts will be required to confirm this observation.

Two-locus genotype combination analysis further characterized the multilocus genetic architecture, identifying higher-risk combinations such as FCGR2B–HLA (2–5) and (2–23). These findings are compatible with a polygenic susceptibility framework in which antigen presentation and Fc-receptor–mediated inhibitory signaling may contribute independently and additively to PV risk. Epistasis modeling did not identify evidence for a multiplicative interaction, suggesting that HLA-DRB1 and FCGR2B primarily contribute through additive rather than synergistic effects.

This interpretation is biologically plausible given the known functional roles of these loci: HLA-DRB1 variants influence antigen presentation to CD4⁺ T cells, whereas FCGR2B regulates inhibitory Fc-receptor signaling that can modulate antibody-mediated immune responses.

Genetic risk score (GRS) analyses further demonstrated a clear cumulative genetic effect, with increasing numbers of susceptibility alleles substantially elevating disease risk. The unweighted GRS showed a strong association with PV susceptibility, indicating that individuals carrying a higher number of risk alleles have markedly increased odds of disease. Incorporating variant-specific effect sizes through the weighted GRS improved predictive discrimination compared with the unweighted model. This improvement reflects the heterogeneous contribution of individual loci to disease risk and is consistent with polygenic risk modeling approaches increasingly applied in autoimmune diseases such as type 1 diabetes and autoimmune thyroid disease (Chatterjee et al. 2016).

These findings indicate that the combined effects of HLA-DRB1 and FCGR2B variants may contribute to disease susceptibility within a cumulative genetic framework. The strong discriminative performance observed for the weighted GRS model (AUC ≈ 0.89) and the XGBoost classifier (AUC ≈ 0.88) suggests that integrating genetic information can improve risk modeling approaches in research settings. Nevertheless, prospective validation in independent and ethnically diverse cohorts will be required before clinical implementation can be considered.

Importantly, the weighted genetic risk score (GRS) was both derived and evaluated within the same dataset, which may lead to optimistic estimates of predictive performance. This internal derivation may inflate discrimination metrics and limit generalizability. Therefore, these findings should be interpreted with caution, and independent external validation is required to confirm the robustness and clinical applicability of the proposed genetic risk model.

The integration of explainable artificial intelligence (XAI) further strengthened these conclusions. The XGBoost model achieved strong classification performance with high sensitivity, indicating robust detection of disease cases. SHAP-based interpretability analysis identified HLA_risk_allele as the dominant driver of model predictions, while FCGR2B_C_carrier contributed a smaller but detectable effect. In contrast, the interaction term showed minimal contribution, supporting the regression-based finding that strong epistatic effects between these loci are unlikely. By quantifying the contribution of each predictor, SHAP provided transparent insight into model behavior, addressing a common limitation of traditional “black-box” machine learning approaches.

Although model discrimination was strong, we applied internal validation procedures to enhance robustness and minimize the risk of overfitting. Nonetheless, independent external validation will be necessary to confirm generalizability before clinical translation can be considered.

Functional annotation analyses were performed to provide biological context for the identified genetic associations. Gene Ontology (GO) and KEGG annotations were examined to place the analyzed genes within known immunological processes. However, because the present study focused on a limited number of candidate genes, these annotations should be interpreted primarily as biological context rather than as evidence of formal pathway enrichment.

HLA-DRB1 plays a central role in antigen presentation through the major histocompatibility complex (MHC) class II pathway, which is essential for activating CD4⁺ T-cell responses. FCGR2B encodes an inhibitory Fc-gamma receptor that regulates B-cell activation and antibody-mediated immune responses. Accordingly, the functional annotations considered in this study are consistent with well-established immunological mechanisms involved in autoimmune blistering diseases, including antigen presentation and Fc-receptor–mediated immune signaling.

Importantly, these analyses were not intended to identify novel biological pathways but rather to provide functional context supporting the observed genetic associations between HLA-DRB1, FCGR2B, and pemphigus susceptibility. Previous studies have similarly highlighted the central roles of HLA-mediated antigen presentation and immune-regulatory signaling pathways in the pathogenesis of pemphigus and related autoimmune blistering diseases.

Therefore, the functional annotation results should be interpreted as supportive biological context aligned with existing immunological knowledge, rather than as independent findings from large-scale pathway enrichment analyses.

A notable strength of this study is the availability of a genotyped cohort of 286 individuals, including 86 biopsy- and serology-confirmed PV cases. This dataset enabled robust evaluation of HLA-DRB1 effects and allowed integrated analyses incorporating two-locus genotype combinations, multilocus modeling, and exploratory machine-learning approaches. The simultaneous availability of HLA-DRB1 and FCGR2B genotypes within the same cohort also provides an analytical depth that has rarely been examined in PV genetic studies.

Several limitations should also be acknowledged. Regression models were not adjusted for demographic covariates, although no statistically significant differences in baseline age or sex distribution were observed between cases and controls. Residual confounding therefore cannot be completely excluded. In addition, genome-wide ancestry markers were not available, preventing formal assessment of population stratification (e.g., principal component analysis). However, cases and controls were recruited from the same clinical center and geographic catchment area, which likely reduces the likelihood of substantial population substructure. Future studies incorporating ancestry-informative markers and larger multi-center cohorts will help further validate and extend these findings.

Beyond these considerations, some additional aspects should be noted. The study was conducted within a single cohort, which may limit population-level generalizability, particularly given the well-recognized ethnic variability in HLA allele distributions. In addition, the potential relationship between the identified immunogenetic architecture and disease severity was not explored in the present study. Although our primary objective was to investigate genetic susceptibility, integrating standardized severity indices in future cohorts may clarify whether specific genetic profiles influence not only disease risk but also clinical course and intensity. Such analyses could contribute to improved genotype–phenotype stratification and a more refined understanding of disease heterogeneity in pemphigus vulgaris.

The present study illustrates the value of integrating multiple analytical approaches within a single genetic dataset. While prior investigations have frequently examined HLA and non-HLA genes independently, the current analysis combined single-locus modeling, two-locus genotype combination analysis, epistasis testing, genetic risk scoring, and SHAP-based model interpretability within a unified analytical framework. This integrated strategy provides complementary perspectives on the immunogenetic architecture of pemphigus vulgaris and may help guide future studies incorporating larger cohorts and broader genomic or multi-omics data.

Overall, the findings suggest that pemphigus vulgaris susceptibility may be shaped by an additive immunogenetic architecture in which HLA-DRB1 represents the primary genetic determinant, while FCGR2B may contribute secondary modulatory effects. The convergence of statistical modeling and machine-learning analyses is consistent with an interpretation involving antigen presentation and Fc-receptor–mediated immune regulation. This integrative perspective may help inform future research aimed at improved immunogenetic characterization and risk modeling in pemphigus vulgaris.

## Conclusion

This study provides an integrated evaluation of how HLA-DRB1 and FCGR2B variants contribute to pemphigus vulgaris susceptibility. The findings reaffirm HLA-DRB1 as the primary genetic determinant, with the *04:02 and *14:01 alleles conferring strong risk and *11:01 and *16:01 demonstrating protective effects. Although the FCGR2B variant showed a modest signal in single-locus analyses, it did not remain statistically significant after multiple-testing correction and should therefore be interpreted cautiously within the broader immunogenetic framework.

Explainable machine learning analyses further indicated that these genetic predictors can be incorporated into classification models with interpretable feature contributions. Functional annotation and protein–protein interaction analyses provided biological context consistent with known immune processes involving antigen presentation and Fc-receptor–mediated immune regulation.

Taken together, the results support an additive immunogenetic framework in which HLA-DRB1 represents the primary susceptibility locus, while FCGR2B may represent a potential modulatory component of immune regulation. Future studies incorporating broader gene panels, multi-omics data, and multi-ethnic cohorts will be important to further validate these observations and improve understanding of the genetic architecture of pemphigus vulgaris.

## Supplementary Information

Below is the link to the electronic supplementary material.

Supplementary Material 1

## Data Availability

The genotype, clinical, and laboratory data used in this study are subject to institutional and regulatory restrictions. These data contain sensitive human participant information and can only be shared upon obtaining the necessary approvals from the relevant third-party institutions, including Karadeniz Technical University and affiliated Farabi Hospital. Therefore, the datasets are not publicly available. De-identified data or specific variables may be shared by the corresponding author upon reasonable request and following formal authorization by the appropriate institutional bodies.

## Appendix

**Table 4:** Primer sequences and amplicon sizes used for FCGR2B genotyping

*Gene*	*Primer ID*	*Primer sequence (5′–3′)*	*Amplicon size (bp)*	*Purpose*
FCGR2B	2281	TAGGACATAGCATTGGATGGG	3579	Exon 5 (Long PCR)
FCGR2B	2282	CAGAAACCAGCAGTAAGGATC	3579	Exon 5 (Long PCR)
FCGR2B	2281	TAGGACATAGCATTGGATGGG	1235	Exon 5 (Nested PCR)
FCGR2B	2236	AGCTCCCCCTGCTCAGAGGCT	1235	Exon 5 (Nested PCR)
FCGR2B	1914	ACAATACGGGCCTAGATCTGAA	—	Sequencing primer

**Table 5:** HLA-DRB1 allele counts and group-specific frequencies in PV cases and controls

*Allele*	*PV (n)*	*PV (%)*	*Control (n)*	*Control (%)*
04:02	66	38.4	18	4.5
14:01	30	17.4	17	4.3
11:04	9	5.2	39	9.8
07:01	7	4.1	37	9.3
01:01	6	3.5	23	5.8
04:03	6	3.5	9	2.3
11:01	6	3.5	39	9.8
13:01	6	3.5	19	4.8
13:02	6	3.5	12	3
03:01	5	2.9	26	6.5
15:01	5	2.9	33	8.3
04:04	4	2.3	4	1
08:03	3	1.7	7	1.8
14:04	3	1.7	0	0
01:02	2	1.2	3	0.8
10:01	2	1.2	11	2.8
04:01	1	0.6	16	4
08:04	1	0.6	2	0.5
11:03	1	0.6	6	1.5
15:02	1	0.6	12	3
08:01	1	0.6	0	0
11:11	1	0.6	0	0
04:05	0	0	6	1.5
04:06	0	0	1	0.3
04:07	0	0	3	0.8
04:08	0	0	5	1.3
08:02	0	0	2	0.5
09:01	0	0	8	2
12:01	0	0	2	0.5
13:03	0	0	12	3
13:05	0	0	1	0.3
16:01	0	0	26	6.5
16:02	0	0	1	0.3
**Total alleles**	172	100	400	100

Frequencies were calculated based on total allele counts within each group (PV: *n* = 172 alleles; Controls: *n* = 400 alleles). Alleles observed fewer than three times in either group were not interpreted individually in regression modeling to avoid unstable effect size estimation

**Table 6:** Numeric coding scheme for two-locus FCGR2B–HLA-DRB1 allele combinations

***Code***	***FCGR2B Allele***		
1	C		
2	T		
**Code**	***HLA-DRB1 Allele***	**Code**	***HLA-DRB1 Allele***
1	01:01	18	10:01
2	01:02	19	11:01
3	03:01	20	11:03
4	04:01	21	11:04
5	04:02	22	11:11
6	04:03	23	12:01
7	04:04	24	13:01
8	04:05	25	13:02
9	04:06	26	13:03
10	04:07	27	13:05
11	04:08	28	14:01
12	07:01	29	14:04
13	08:01	30	15:01
14	08:02	31	15:02
15	08:03	32	16:01
16	08:04	33	16:02
17	09:01		

**Table 7:** XGBoost hyperparameter settings used in the study

*Parameter*	*Value*
Objective	binary: logistic
Eval_metric	auc
Max_depth	3
Eta (learning rate)	0.1
Subsample	1.0
Colsample_bytree	1.0
Scale_pos_weight	2.14
Nrounds	200
Train/test split	70/30
Random seed	123

**Table 8:** Genotype–phenotype association results for FCGR2B c.671 T>C (I232T) polymorphism

*Allele / Model*	*Test*	*Odds Ratio(OR)*	*95% CI*	*p-Value*	*FDR (q)*
c.671 T > C (C-carrier, CC + CT vs. TT)	Fisher’s Exact Test	1.63	0.83–3.15	0.138	0.117
c.671 T > C (C-carrier, CC + CT vs. TT)	Firth Logistic Regression	1.64	0.88–3.01	0.117	0.117

Analyses were conducted under a dominant genetic model (C-allele carrier, CC + TC vs. TT). Although the direction of effect suggested an increased risk for C-allele carriers, neither Fisher’s exact test nor Firth logistic regression reached statistical significance (*p* > 0.05). No associations remained after false discovery rate (FDR) correction

**Table 9:** Multivariable logistic models for HLA-DRB1 and FCGR2B (with interaction) in pemphigus vulgaris

*Model*	*Term*	*Odds Ratio (OR)*	*95% CI*	*p-value*	*q (FDR)*	*AUC*	*C-stat*	*Somers’ D*	*Brier*
*Model 1: FCGR2B*	FCGR2B_C_carrier	1.64	0.88–3.02	0.117	0.117	0.54	0.54	0.079	0.208
*Model 2: HLA*	HLA_risk_allele	17.65	9.13–36.94	< 0.001	< 0.001	0.80	0.80	0.602	0.146
*Model 3: Combined*	FCGR2B_C_carrier	1.53	0.72–3.23	0.267	0.267	0.81	0.81	0.626	0.145
	HLA_risk_allele	17.29	8.95–36.15	< 0.001	< 0.001	0.81	0.81	0.626	0.145
*Model 4: Interaction*	FCGR2B_C_carrier	2.16	0.50–7.64	0.274	0.412	0.81	0.81	0.626	0.145
	HLA_risk_allele	17.77	8.86–44.39	< 0.001	< 0.001	0.81	0.81	0.626	0.145
	FCGR2B_C_carrier × HLA_risk_allele	0.63	0.14–3.36	0.570	0.570	0.81	0.81	0.626	0.145

*NOR* odds ratio; *CI* confidence interval; *q (FDR)* Benjamini–Hochberg false discovery rate–adjusted p; *AUC* area under the ROC curve; *C-stat* concordance index; *Somers’ D* rank correlation between predicted and observed outcomes; *Brier* mean squared prediction error
