# Supplementary material for: Genetic and immunological determinants of Pemphigus vulgaris: integrative analysis of HLA-DRB1 and FCGR2B variants
Source: Immunogenetics. 2026 May 20;78(1):9. doi: 10.1007/s00251-026-01402-5 (PMC13186795; doi:10.1007/s00251-026-01402-5)
Supplement: Supplementary file 1 — Supplementary Material 1 [file 251_2026_1402_MOESM1_ESM.pdf]

Supplementary Methods and Raw Molecular Data

Supplementary Methods S1. Genotyping procedures

Genomic DNA was extracted from peripheral blood samples using standard salting-out procedures. HLA-DRB1 alleles were genotyped using a PCR-SSO (sequence-specific oligonucleotide probe) Luminex-based method (LABType SSO, One Lambda, Thermo Fisher Scientific), following the manufacturer's instructions. Biotinylated PCR products were hybridized to allele-specific oligonucleotide probes immobilized on fluorescently coded microspheres and detected using streptavidin-phycoerythrin. Fluorescence signals were acquired on a LABScan3D system (One Lambda), and allele assignment was performed using HLA Fusion software.

FCGR2B genotyping was performed using PCR-based methods, and allelic variants were confirmed by sequence analysis where applicable.

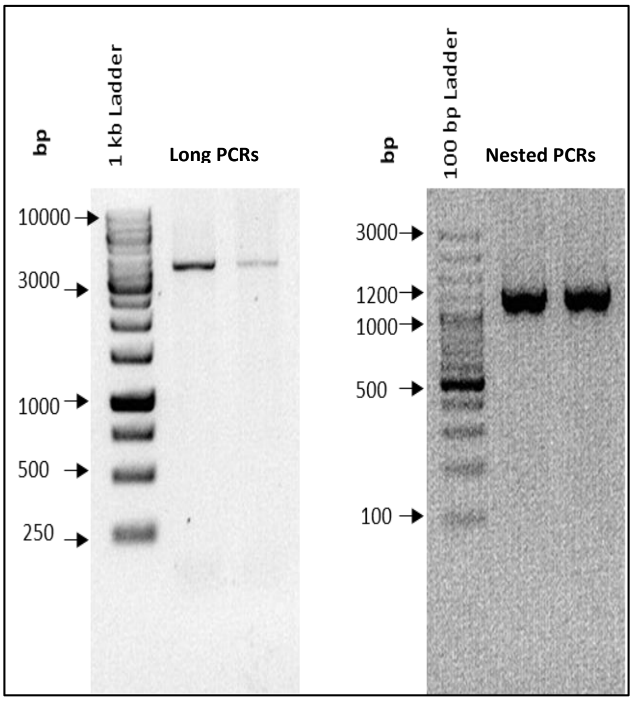

Supplementary Figure S1. Uncropped agarose gel electrophoresis image showing PCR amplification products used for genotyping analyses. Molecular weight markers and all sample lanes are shown.

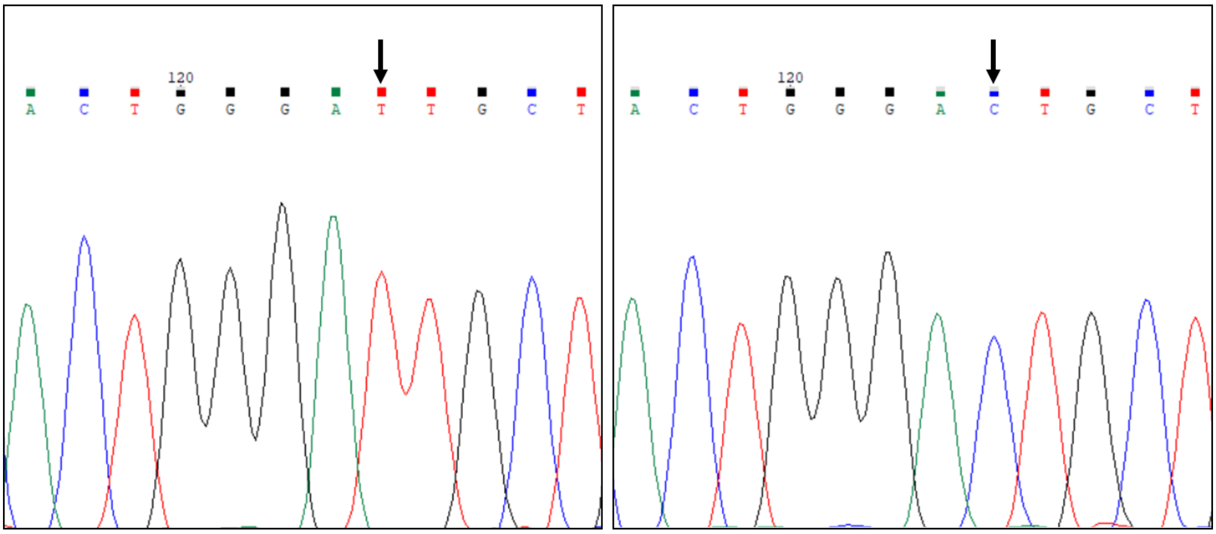

Supplementary Figure S2. Representative Sanger sequencing chromatograms illustrating nucleotide variants used for confirmation of *FCGR2B* genotypes. Arrows indicate the polymorphic positions.

We would like to clarify that **no Western blot or immunoblot analyses were performed** in this study. All molecular analyses were conducted at the DNA level and focused on genetic variation and immunogenetic susceptibility. **Accordingly, protein-level assays and blots are not applicable to the present work.**

To ensure transparency, we have provided uncropped agarose gel images of PCR products and representative sequencing chromatograms as Supplementary Figures, along with detailed genotyping methodology.
